# Analysis of Micro- and Nano-Structures of the Corneal Surface of *Drosophila* and Its Mutants by Atomic Force Microscopy and Optical Diffraction

**DOI:** 10.1371/journal.pone.0022237

**Published:** 2011-07-21

**Authors:** Michail Kryuchkov, Vladimir L. Katanaev, Gennadiy A. Enin, Anton Sergeev, Alexander A. Timchenko, Igor N. Serdyuk

**Affiliations:** 1 Institute of Protein Research, Russian Academy of Sciences, Pushchino, Russian Federation; 2 Department of Pharmacology and Toxicology, University of Lausanne, Lausanne, Switzerland; University of South Florida College of Medicine, United States of America

## Abstract

*Drosophila melanogaster* is a model organism instrumental for numerous biological studies. The compound eye of this insect consists of some eight hundred individual ommatidia or facets, ca. 15 µm in cross-section. Each ommatidium contains eighteen cells including four cone cells secreting the lens material (cornea). High-resolution imaging of the cornea of different insects has demonstrated that each lens is covered by the nipple arrays - small outgrowths of ca. 200 nm in diameter. Here we for the first time utilize atomic force microscopy (AFM) to investigate nipple arrays of the *Drosophila* lens, achieving an unprecedented visualization of the architecture of these nanostructures. We find by Fourier analysis that the nipple arrays of *Drosophila* are disordered, and that the seemingly ordered appearance is a consequence of dense packing of the nipples. In contrast, Fourier analysis confirms the visibly ordered nature of the eye microstructures - the individual lenses. This is different in the *frizzled* mutants of *Drosophila*, where both Fourier analysis and optical imaging detect disorder in lens packing. AFM reveals intercalations of the lens material between individual lenses in *frizzled* mutants, providing explanation for this disorder. In contrast, nanostructures of the mutant lens show the same organization as in wild-type flies. Thus, *frizzled* mutants display abnormal organization of the corneal micro-, but not nano-structures. At the same time, nipples of the mutant flies are shorter than those of the wild-type. We also analyze corneal surface of glossy-appearing eyes overexpressing Wingless - the lipoprotein ligand of Frizzled receptors, and find the catastrophic aberration in nipple arrays, providing experimental evidence in favor of the major anti-reflective function of these insect eye nanostructures. The combination of the easily tractable genetic model organism and robust AFM analysis represents a novel methodology to analyze development and architecture of these surface formations.

## Introduction

Model organisms are powerful tools to study biological phenomena, especially when similar investigations on human beings are impossible due to technical and ethical aspects. One of the most popular model organisms is the fruit fly *Drosophila melanogaster* [1]. The compound eye of this insect provides a useful system for morphological inspection of various mutations affecting development of this organ, and has served to uncover several developmental mechanisms playing ubiquitous roles in animal, including human, development [2], [3].

**Figure 1:**
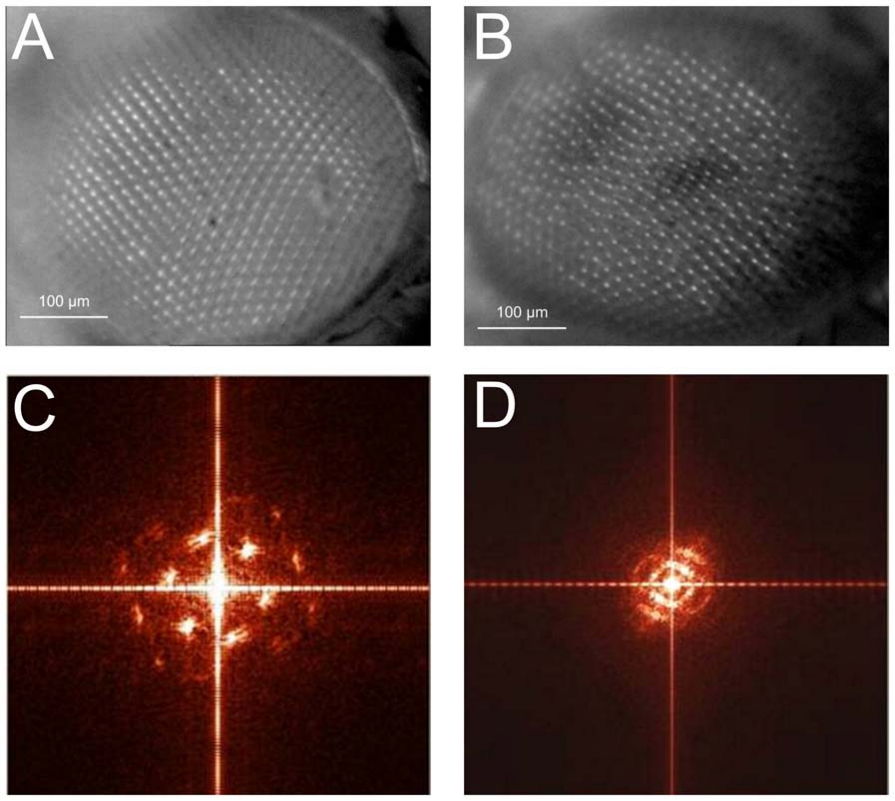
Fourier transformation of *Drosophila* eye optical images confirms lack of order in ommatidial arrangement in *frizzled* mutants. Optical images of the *Drosophila* eyes of the wild-type (A) and *frizzled* mutant (B) genotype were detected with a digital optical microscope. Fourier-transformation of the images confirms order in ommatidial arrangement in wild-type (C), but not mutant (D) eyes.

Up to now, most studies were devoted to the analysis of the eye microstructure, i.e. composition of the ommatidia (facets) and their histological cross-section characterization [4]. A *Drosophila* ommatidium contains eighteen cells, including eight photoreceptors, pigment cells, cells of the mechanosensory protective bristle, and four cone cells secreting the lens material. The individual lenses of the adult eye are hexagonal in shape; the lens hexagons are neatly packed in the insect eye in a crystalline order (Fig. 1A). This outer appearance is reflected by the inner organization of the ommatidia. The six outer photoreceptors of each facet form in cross-section a chiral trapezoid; orientation and chirality of these trapezoids are uniform in each hemisphere of the eye and are mirror-reflected in the other hemisphere [4]. This phenomenon is known as planar cell polarity (PCP), whereas cells of the epithelial origin, in addition to being polarized in the “vertical” apico-basal direction, additionally display polarization in the “horizontal” plane of the tissue [5]. PCP is a wide-spread phenomenon found in insects as well as vertebrates [5], [6]. The molecular control over PCP establishment is mediated by a cascade of protein-protein interactions, initiated by the transmembrane protein Frizzled [5], [7], a member of the G protein-coupled receptor superfamily [8], [9]. Mutations in the frizzled gene, or other genes participating in the PCP, result in randomization of the ommatidial chiral forms and orientation [10], [11], which leads to the disorganized external appearance of the *Drosophila* eye [10], [11], [12], often referred to as the “rough eye” phenotype (Fig. 1B).

The ligand interacting with Frizzled in *Drosophila* PCP signaling is still unknown. In contrast, the secreted lipoglycoprotein Wingless (Wg) acts as the ligand for Frizzled receptors in another type of intracellular signaling cascade called canonical or β-catenin signaling [13]. The canonical Wg/Frizzled signaling controls developmental cell fates and is implicated in human carcinogenesis [14]. This pathway plays multiple roles in *Drosophila* eye formation [3], [15], including the late stages of cone cell development [16]. A dominant Wg allele called *Glazed* was identified by Thomas Morgan 75 years ago, and causes loss of photoreceptor cells through pigment cell-derived misexpression of Wg [17]. Similar phenotypes emerge from late overexpression of Wg in the postmimotic eye cells with the *GMR* enhancer [18], cone and primary pigment cells with the *sparkling* enhancer [16], or in a subset of photoreceptor and cone cells with the *sevenless* enhancer [17]. In all cases, as suggested by the allele name *Glazed*, the *Drosophila* eye obtains a glossy appearance, suggesting cone cell and lens defects [16]. However, unlike the massive photoreceptor cell loss in Wg-overexpressing eyes, only occasional loss of one or two cone cells from the normal four-cell cluster can be seen [16], [17].

In contrast to this microstructural analysis, information about the fine structures of the corneal surface of the *Drosophila* eye is relatively scarce. Scanning electron microscopy has been applied to visualize the so-called nipple arrays: nanometer-scale evaginations of the corneal surface [19], [20]. They originate from secretion of the lens material by the regularly spaced microvilli of the cone cells [20], [21]. These evaginations have been extensively studied in moths and butterflies by means of electron and atomic force microscopy (AFM) [22], [23], [24], [25], [26]. These nipples, typically ca. 200 nm in height and spacing, are believed to be arranged in a crystalline hexagonal pattern and, being smaller than the wavelength of the visible light, have been proposed to serve the antireflective function [27], [28]. This idea inspired development of artificial anti-reflective “moth-eye” coating applications [28]. However, direct experimental evidence for the anti-reflective function of insect nipple arrays has been lacking, and other functions of these nanostructures might also be expected, for example the anti-wetting or self-cleaning function known as the Lotus effect [28], [29].

Although nipple arrays of some insects are well-characterized morphologically, the molecular mechanisms governing their formation are elusive. What drives formation of apparently crystalline-ordered 200 nm-high nipples of the butterflies and moths [22], [24] *vs.* shorter nipples fused into ridges in some dipterans [25], [30] is unknown. Knowledge over the molecular mechanisms governing formation of the nanometer-scale corneal evaginations could permit formation of nipple arrays with novel/desired properties. Subsequent investigation of the anti-reflective or anti-wetting characteristics of such “constructed” nipple arrays may have potential technological applications. The use of a genetically tractable insect is clearly needed to address these issues. So far, the nipple arrays of *Drosophila melanogaster* have not been systematically analyzed, nor was the effect of any mutations on their formation.

In this paper we for the first time present images of the ommatidial external surface of the *Drosophila* fruit fly obtained by AFM at high resolution (ca. 20 nm). We perform a detailed analysis of the images and their Fourier-transforms at the micro- and nano-level resolution. At the micro-level, we demonstrate the clear differences between the wild-type and *frizzled* mutant flies to be a morphological consequence of non-regular incorporations of the lens material between ommatidial lenses in *frizzled* animals. At the nano-scale, we show the dimensions of the nipples covering the corneal surface of *Drosophila* to be 250 nm in cross-section and 30 nm in height. We find these nipples to be densely packed in a chaotic manner, with small areas of hexagonal arrangement both for the wild-type and *frizzled* mutant lines. Additionally, we find that nipples of the *frizzled* flies have a somewhat decreased height. Analysis of the glossy Wg-overexpressing eyes shows a dramatic loss of nipple structures, offering the mechanistic explanation for this phenotype first described in 1936, and serving as a first experimental evidence for the anti-reflective function of insect nipple arrays.

Our results highlight the effectiveness of AFM and optical diffraction to analyze the effect of mutations on the eye architecture of *Drosophila*, and open the way to the systematic investigation of the mechanisms of nipple array formation through the full power of *Drosophila* genetics.

## Results

To analyze *Drosophila* cornea and their nipple arrays, we utilized atomic force microscopy (AFM), optical diffraction, and Fourier transformation. We used these methods to characterize cornea of wild-type eyes and those of mutant flies. The *frizzled* mutation was selected as a first attempt to study the genetic influence on nipple formation, as other insects’ nipple arrays are reported to form crystalline order [22], [24] not dissimilar to the crystalline order of the micro-scale ommatidial organization which is under the Frizzled-controlled planar cell polarity (PCP) signaling control [5], [7]. Thus, we argued that perhaps the *frizzled* mutation might affect both the micro-scale ommatidial and the nano-scale nipple order of *Drosophila* cornea. As the methods of our investigation represent a novel approach to study *Drosophila* eye surfaces, we decided to utilize them in a gradual increase in resolution from the micro-scale to the nano-scale level.

Our AFM with the built-in digital optical microscope permits to study the objects in a wide range of dimensions from millimeters to tens of nanometers. We first analyzed the microstructure of *Drosophila* eye surface with the optical microscope. In Fig. 1 digital images of the eye surface are presented for the wild-type (1A) and *frizzled* mutant (1B) flies. Visual inspection of the patterns obtained identifies essential differences in the ommatidial packing: regular for the wild-type and “rough” for the mutant eyes, as has been previously reported [12]. More detailed information can be extracted from analysis of two-dimensional Fourier spectra of the presented images. The Fourier transforms of the wild-type eye images display reflexes up to the fourth order, arranged in the hexagon apexes reflecting periodic hexagonal ommatidial packing (Fig. 1C). A certain degree of smearing of the reflexes can be explained by the surface curvature (Fig. 1A). For the *frizzled* mutant line the observed “rough eye” effect (Fig. 1B) is well reflected in the two-dimensional Fourier transform of the image, where no distinct reflexes and instead a set of concentric circles reflecting a non-ordered arrangement can be seen (Fig. 1D). However, more intense regions arranged as apexes of hexagon, reflecting existence of small regions of the eye surface with the dense hexagonal ommatidial packing, can also be seen in the Fourier spectrum of *frizzled* eyes (Fig. 1D). Thus, analysis of the Fourier transforms of the external appearance of wild-type and *frizzled* mutant eyes confirms the well-established data on the *Drosophila* eye analysis by electron microscopy, whereas the normal hexagonal shape and dense packing of ommatidia in a periodic two-dimensional grid are disturbed upon mutations in the frizzled gene [4], [12].

**Figure 2:**
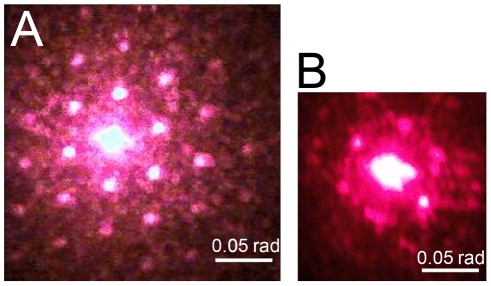
Diffraction patterns of *Drosophila* cornea confirm lack of order in ommatidial arrangement in *frizzled* mutants. Corneal preparations from wild-type (A) and *frizzled* mutant (B) eyes were irradiated with a laser beam of 630 nm to collect diffraction patterns.

The existence of regularity in the arrangement of wild-type ommatidia permits application of the method of optical diffraction to identify the packing mode without any mathematical data treatment. To perform such experiments, a region of wild-type fly cornea was irradiated by a laser beam. The registered diffraction pattern with sufficiently many reflexes (up to the fourth order) confirms the periodic hexagonal packing of wild-type ommatidia (Fig. 2A). At the same time, the diffraction pattern from the cornea of the *frizzled* mutant is noticeably smeared and only reflexes of the first order arranged in the hexagon apexes are observed (Fig. 2B). This confirms the limited periodicity of ommatidial arrangement and existence of only small regions with dense hexagonal packing of ommatidia in the mutant flies. Ommatidial lens dimensions can be estimated from the diffraction pattern at small diffraction angles according to the formula:
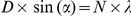
where *D* is the period of packing of the elements, α is the diffraction angle, λ is the irradiation wavelength, and *N* is the order of diffraction. The calculated dimension of an ommatidial lens is 13 µm both for the wild-type and mutant lines, which is close to the values previously obtained by other methods [4].

**Figure 3:**
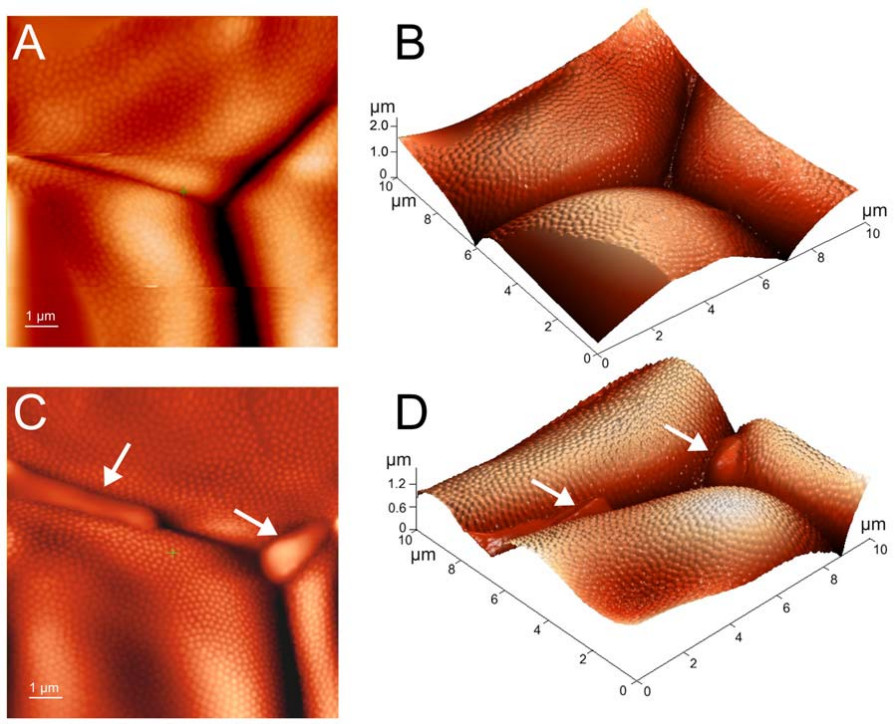
Fine structure AFM images of *Drosophila* ommatidial surface reveal irregularities in the lens material deposition in *frizzled* mutants. Corneal surface of the wild-type (A, B) and *frizzled* mutant (C, D) eyes was analyzed at high resolution with AFM. Field of view is 10×10 µm. Arrows indicate intercalations of the lens material between ommatidial lens borders in the *frizzled* mutant (C, D). (A, C) represent top views, while (B, D) are their three-dimensional representations.

As it is the packing and not the size of ommatidial lenses which becomes aberrant in the *frizzled* flies, we decided to further investigate the packing details at the micro-scale by AFM. Fig. 3 depicts images of the fine structure of ommatidia from the wild-type and *frizzled* mutant flies. The interface between ommatidia can be clearly seen in the AFM images of the 10 µm scale. This level of resolution permits understanding of the reason for the distortion in ommatidial lens packing of the mutant flies described above. While the borders of the wild-type ommatidia are tightly aligned to each other (Fig. 3A, B), irregular infiltrations of the lens material fill the gaps between lenses of the *frizzled* mutants (Fig. 3C, D). These infiltrations indicate that the packing of the mutant lenses is less compact, making them more loosely aligned to each other, explaining the “rough” appearance and the lack of regularity described above.

**Figure 4:**
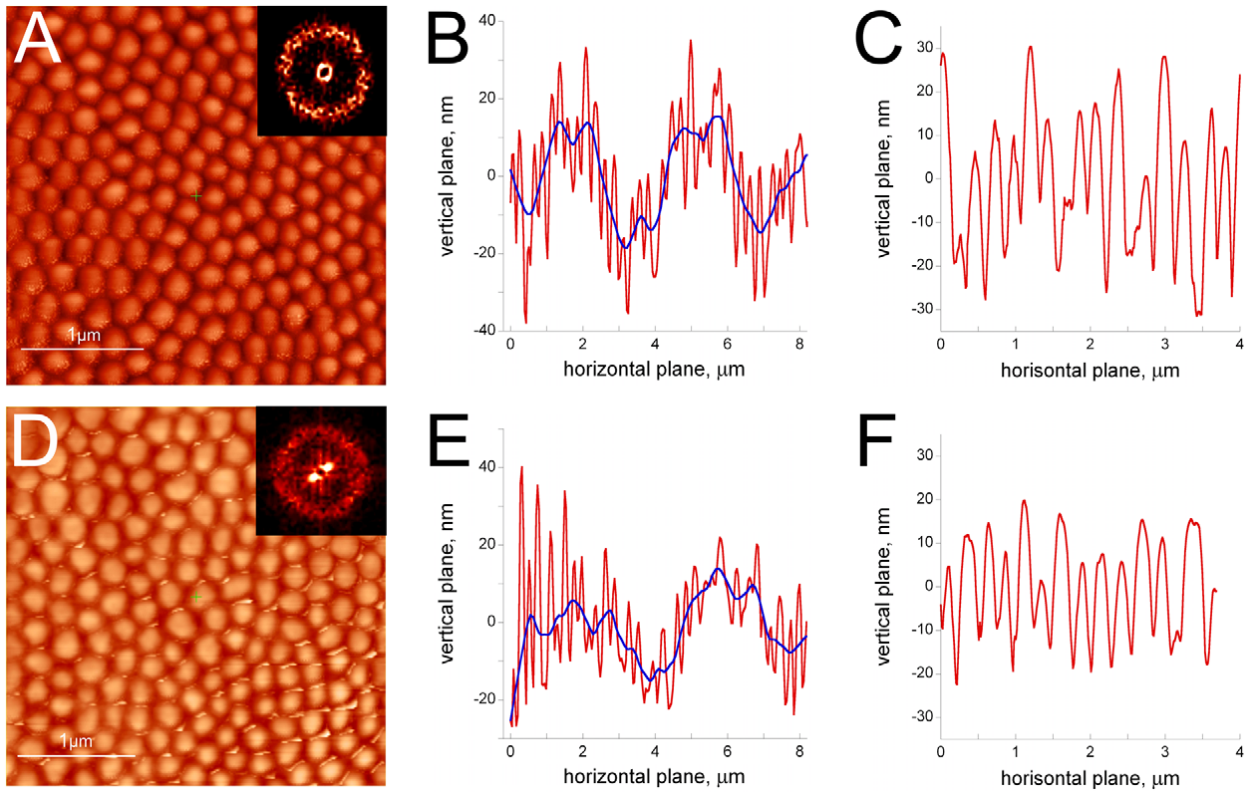
High-resolution analysis of the *Drosophila* nipple arrays. Corneal surface of the wild-type (A) and *frizzled* mutant (D) eyes was analyzed at high resolution with AFM. Field of view is 3×3 µm. Fourier transform spectra of the AFM images are shown as inserts in (A, D). (B, E) are cross-sectional profiles of representative scans of wild-type (B) and *frizzled* mutant (E) cornea of ca. 8 µm length. Blue lines in (B, E) are smoothing curves of the height recording curves depicted with the red lines. (C, F) are representative cross-sectional 4 µm-long profiles of flat areas of wild-type (C) and *frizzled* mutant (F) cornea such as those on (A, D).

Interestingly, the corneal surface of individual lenses does not appear to be ideally curved. Instead, elevations of roughly 4 µm in width and ca. 40 nm in height could be seen in the AFM images (Fig. 3) and the cross-section profiles (see Fig. 4B, E as examples). Since the lens has ca. 13 µm in cross-section and is a product of secretion of four cone cells [4], we hypothesize that these irregularities in the corneal surface height may represent portions of the lens produced by the individual cone cells.

We continued to increase the resolution of the surface analysis of the wild-type and mutant eyes of *Drosophila*. Fig. 4A, D depicts AFM images of the fine structure of the ommatidium surface obtained at the 3 µm scale. Surface of the individual lens of each ommatidium is covered with the array of nipples, the cross-section of nipples being roughly 250 nm (Fig. 4A, D). Careful determination of the nipple broadness measured as the distance from the tip of a nipple to the tip of the next one is measured as 255±5 nm for the wild-type cornea, and 251±5 nm for the *frizzled* mutant cornea (mean ± sem, n>150 nipples analyzed in seven independent readings of different corneal preparations, Fig. 4C, F), which is somewhat broader than described previously with other methods [20]. While electron microscopy studies have previously established that the fly nipple arrays are considerably shorter than those of moths and butterflies [20], [23], the exact determination of the height of *Drosophila* nipples has been missing. Using the AFM technique, we measure this height as ca. 30 nm, which makes it 6–7 times shorter than that of many Lepidopterans [23], [24]. The height of the wild-type nipples is measured as 31.1±1.3 nm, while that of the *frizzled* mutants nipples is 26.4±1.2 nm (mean ± sem, n>150 nipples analyzed in seven independent readings of different corneal preparations, Fig. 4C, F). Thus, the *frizzled* mutation does not affect the broadness of nipples, but makes them shorter by ca. 5 nm, or by 15% of the initial height. This difference, albeit small, is statistically significant (P value by the unpaired t-test being 0.00044).

Visual inspection of the images does not identify clear regularity in the nipple arrangement both for the wild-type and the *frizzled* mutant lens (Fig. 4A, D), although small regions with an apparently hexagonal packing of the nipples could be found, as corroborated by the nipple array analysis in moths and butterflies [24]. For the formal analysis of the presence or absence of order in the *Drosophila* nipple arrays, Fourier transforms of the AFM images were obtained (inserts in Fig. 4A, D). The resulting Fourier spectra reveal no discernable regularity in the nipple arrays for both genotypes. For some regions of the lens of either genotype, the Fourier spectrum could show the shape of a smeared hexagon, indicating existence of small regions in the ommatidium surface with dense hexagonal packing of nipples (data not shown). However, because most regions of the lens, regardless of the genotype, do not show any hexagonal organization in the Fourier spectra (inserts in Fig. 4A, D), we conclude that nipple arrays of *Drosophila* lens are disordered, and that mutations in the frizzled gene do not affect organization of the nano-scale lens surface structures.

**Figure 5:**
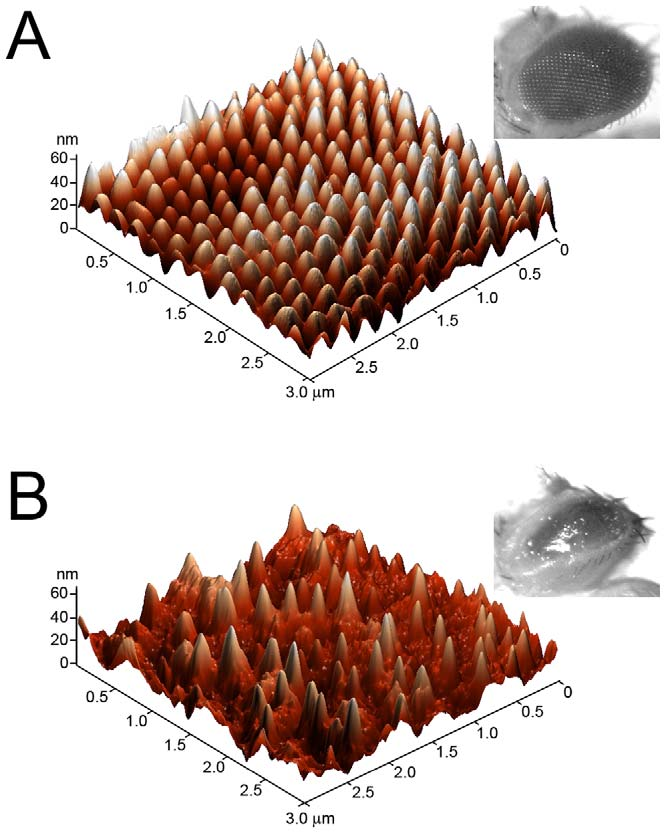
Overexpression of Wg leads to a dramatic loss of nipple arrays, correlating with the glossy eye phenotype. Three-dimensional AFM representation of nipple arrays of wild-type flies (A) and the *GMR-Gal4; UAS-Wg* flies overexpressing Wg in postmitotic eye cells (B). A catastrophic loss of nipples is observed upon Wg overexpression, with few remaining nipples randomly spaced with huge gaps between them. This loss of nipples correlates with the overall glossy appearance of the mutant eyes (B, insert), as opposed to the wild-type eyes (A, insert). The eye size in *GMR-Gal4; UAS-Wg* flies is also reduced due to photoreceptor loss. A light microscope was used to take images of the whole eyes shown in inserts.

As the nipple dimensions are smaller than the wavelength of the visible light, the main function of the insect nipple arrays is predicted to be antireflective [28]. A mechanical model studying insect nipples predicts that they increase the transmission of visible light through the lens by ca. 4%, which corresponds to a reduction in reflectance by 10-to-100 fold [31]. However, nipple arrays have been traditionally analyzed in insect species which are not genetically tractable and direct experimental evidence in favor of the anti-reflective function could not be provided. We decided to address this issue in *Drosophila*, for which several mutant lines exist with the glossy appearance of the eye. These are the lines *e.g.* overexpressing the lipoglycoprotein Wg (compare inserts on Fig. 5A and 5B) which serves as the ligand for Frizzled receptors in the canonical β-catenin-dependent signaling pathway [13]. The general size of the eye in these lines is reduced (see insert in Fig. 5B) due to loss of photoreceptor cells, whereas pigment and cone cells remain [16], [17]. Analysis of the nipple arrays of the *GMR-Gal4; UAS-Wg* line showed a catastrophic loss of nipples; the remaining nanostructures are randomly spaced with large gap areas (Fig. 5B). Thus, we show that the glossy appearance of *Drosophila* eyes correlates with the loss of nipple arrays, providing the first experimental evidence (although circumstantial) for the major anti-reflective function of insect nipple arrays.

## Discussion

Our data for the first time combine physical methods, such as AFM and optical diffraction, mathematical analysis, and genetic approach to study the fine structures of the cornea of the genetically tractable model insect *Drosophila melanogaster*. Such combination of these powerful techniques paves the way to systematic investigation of mechanisms and properties of the nano-scale nipple arrays of the insect lens. Furthermore, easiness of genetic manipulations of this fruit fly permits future synthetic biology approaches to *e.g.* construct modified/engineered nipple arrays and characterize their physical properties as anti-reflective or anti-wetting coatings. Such studies may not only provide insights into the biological mechanisms of vision, but also inspire potential industrial developments.

The power of our approach is illustrated by the analysis of the glossy eye surfaces of Wg-overexpressing flies (Fig. 5B). Such mutants were first isolated by the founder of *Drosophila* genetics Thomas Morgan in 1936, and actively studied in the recent decades [17]. Overexpression of Wg results in a massive loss of photoreceptors in these eyes. In contrast, pigment cells remain intact, and only occasional cone cell loss occurs [16], [17]. Although it was clear that some defects in cone cells secreting the lens were behind the glossy appearance of these mutant eyes [16], no mechanistic understanding was provided. Our analysis shows the dramatic loss of the nipple nanostructures on the corneal surface of such eyes (Fig. 5B). This observation not only offers an explanation for the glossy eye phenotypes known for seventy five years, but is also first direct evidence in favor of the 40 years-old idea that the major function of the insect corneal nipple arrays is antireflective.

Additionally, our investigation gives useful hints towards the nature of biological order and disorder. Traditional biology often relies on the visual inspection of biological structures to conclude about their ordered *vs.* disordered nature. Our analysis shows how misleading this simplified approach may be. Indeed, both the microscopic (ommatidial) and nano-scale (nipple arrays) structures of the wild-type flies may suggest their ordered hexagonal crystalline packing, as has been proposed also for other insects [27], [28]. However, formal Fourier analysis unequivocally proves order in the ommatidial packing, but demonstrates lack of it in the nipple arrays. While regions of the seemingly hexagonal packing can be found in *Drosophila* (Fig. 4A) and other insects’ nipple arrays [23], [24], [30], the overall organization of these nanostructures appears disordered, as is confirmed by the Fourier analysis of *Drosophila* nipples (insert in Fig. 4A). In this regard, the pseudo order in the nipple arrays appears as the mere outcome of the dense packing of nipples.

These considerations bring about further important notions concerning biological order formation. Indeed, the Frizzled-initiated planar cell polarity (PCP) signaling appears to have been evolutionary added “on top” of the dense packing-mediated pseudo order in organization of *e.g.* hairs on the insect cuticle. Indeed, mutations in the frizzled gene or genes encoding other components of the PCP pathway do not fully randomize hair orientation in *Drosophila* wings. Instead, patches of the ordered hair orientation, separated by swirls or whorls, are formed [12], [32]. This pseudo order likely results from the dense cellular packing [33], [34] and can also be observed in other organisms in the absence of PCP [35]. Interestingly, it can also be recapitulated by mechanical models, such as the two-dimensional population of densely-packed metal rods under vibration [36]. It also strongly resembles the pseudo order of the nipple arrays (Fig. 4). These considerations suggest that sometimes the biological pseudo order achieved by the densely packed cells is the only (or the main) mechanism present behind the apparent uniformity in cellular organizations, arguing against implying a PCP-like mechanism in certain cases such as *e.g.* germ-band elongation - the developmental elongation of the *Drosophila* embryo [37], [38].

We end our article with the following conclusions:

1. The combined application of optical methods (light microscopy and optical diffraction) and AFM permitted us to study the eye surface structure of the fruit fly *Drosophila melanogaster* both at the micro- and the nano-levels.
2. Analysis of the optical images of the eye surface using their two-dimensional Fourier transforms confirmed distortion of ommatidial packing regularity by mutations in the frizzled gene. AFM analysis identifies that the hexagonal ommatidial packing is disturbed in the *frizzled* mutant line through non-regular infiltrations of the lens material between ommatidia, reducing their packing density.
3. For the first time, high-resolution (20 nm) AFM analysis of the ommatidial surface of wild-type and mutant *Drosophila* flies has been performed.
4. The lens surface is not uniformly curved but instead contains “waves” of ca. 4 µm in broadness and 40 nm in height; these irregularities may result from lens secretion by the four individual cone cells of each ommatidium.
5. The surface of the ommatidial lens at the nano-scale represents the array of nipples with cross-section of 250 nm and height of 30 nm. Nipples of the *frizzled* mutant flies are shorter by 5 nm but have the same broadness as those of wild-type flies. Mutations in the frizzled gene influence arrangement of ommatidia at the micro-scale but have no effect on the ommatidium nano-scale structures.
6. A catastrophic loss of nipples is observed in Wg-overexpressing “glazed” eyes, suggesting that the glossy eye appearance in some *Drosophila* mutant lines is due to loss of the anti-reflective nipple arrays.
7. The combination of the physical (*e.g*. AFM) and genetic methods allows future investigations of the mechanisms governing the nipple array formation, as well as creation and characterization of the artificial nipple array nanostructures.

## Materials and Methods

*D. melanogaster yw* (wild-type), *fz^[H51]/^fz^[K21]^* transheterozygous mutant [12], and *GMR-Gal4; UAS-Wg* (Bloomington stock center) lines were raised at 23°C at standard conditions [39]. Male flies were used throughout the experiments. Binocular microscope with a digital camera was used to take whole eye images in Fig. 5.

To prepare corneal samples, the head of an adult *Drosophila* fly was cut out of the body, followed by removal of the mouth apparatus with a scalpel, splitting of the head into two hemispheres, and careful extraction of the brain tissue with forceps. Next, the cornea was cleared from the head capsule tissue as well as the underlying brain material with a scalpel. The sample was flattened by making some peripheral cuts and attached to a glass slide for AFM by means of a two-sided scotch tape. For optical diffraction recordings, the cornea was stabilized between two cover glasses.

AFM scanning of the *Drosophila* lens was performed with the Integra-Vita microscope (NT-MDT, Zelenograd, Russia). For the semi-contact procedure, the nitride silicon cantilever NSG 03 (NT-MDT) was used. The parameters of the cantilever were: length: 100 µm, resonant frequency: 62–123 kHz, radius: 10 nm, force constant: 0.4–2.7 N/m. For the contact procedure, the cantilever CSG 10 (NT-MDT) was used, with the following parameters: length: 250 µm, resonant frequency: 14–28 kHz, radius: 10 nm, force constant: 0.03–0.2 N/m. The choice between the semi-contact and the contact measuring procedures was dictated by the size and curvature of the studied surface of the sample, but provided essentially identical results. In each AFM experiment several scans were made to check the reproducibility of images and the absence of possible surface damages. The “FFT analysis” software tool of the AFM (NT-MDT) was used to obtain two-dimensional Fourier transforms of the images.

In optical diffraction experiments, the diffraction pattern from *Drosophila* corneal samples was obtained by irradiating the cornea stabilized between two cover glasses with the laser beam with the wavelength of 630 nm in the TEM_00_ mode. Since the cross-section dimension of the laser beam (ca. 2 mm) exceeded the size of object, the lens with the focal distance F = 30 cm was used to focus laser radiation on the object. The lens-screen distance was 194 cm.

Nipple height and broadness calculation was performed by the analysis of the cross-section profiles of the scans as those presented on Fig. 3 and 4. Nipple height was calculated as the average distance from the tip of each peak to the bottom to its left and right; nipple broadness was calculated as the distance between the adjacent peaks. 8 µm-long cross-section profiles (Fig. 4B, E) were treated with a smoothing function using the KaleidaGraph 4.02 program (Synergy Software).
